# Genetic evaluation of hyperphenylalaninemia patients with tetrahydrobiopterin deficiency in Iranian population: Identification of four novel disease‐causing variants

**DOI:** 10.1002/mgg3.2081

**Published:** 2022-11-16

**Authors:** Seyedeh Helia Sadat Fatemi, Peyman Eshraghi, Mahmoud Ghanei, Tayebeh Hamzehloei

**Affiliations:** ^1^ Medical Genetics and Molecular Medicine Department, Faculty of Medicine Mashhad University of Medical Sciences Mashhad Iran; ^2^ Medical Genetics Research Center, Faculty of Medicine Mashhad University of Medical Sciences Mashhad Iran; ^3^ Clinical Research Development Unit of Akbar Hospital, Faculty of Medicine Mashhad University of Medical Sciences Mashhad Iran

**Keywords:** 6‐pyruvoyltetrahydropterin synthase, dihydropteridine reductase, novel variant, phenylketonurias, protein modeling

## Abstract

**Background:**

Hyperphenylalaninemia (HPA) is the most common inborn error of amino acid metabolism worldwide. At least 2% of HPA cases are caused by a deficiency in tetrahydrobiopterin (BH4) metabolism. Genes such as *QDPR* and *PTS* are essential in the BH4 metabolism. This study aims to identify disease‐causing variants in HPA patients, which may be helpful in genetic counseling and prenatal diagnosis.

**Methods:**

A total of 10 HPA patients were enrolled in this study. The coding and adjacent intronic regions of *PTS* and *QDPR* genes were examined using Sanger sequencing. Protein modeling was also performed for novel identified variants.

**Results:**

Ten patients and a total of 20 alleles were studied, which led to the identification of 10 different variants. All variants identified in *PTS* and *QDPR* were missense, except for the c.383_407del variant in the *QDPR*. Also, three novel variants were identified in the *QDPR*, including c.79G>T, c.383_407del and c.488G>A, and a novel variant, c.65C>G, in the *PTS*.

**Conclusions:**

Despite the genetic similarities in the disease‐causing variants, differences were observed in the Asian and European populations with our populations; As a result, similar but more extensive studies are needed to investigate the distribution of disease‐causing variants in genes involved in non‐PKU hyperphenylalaninemia.

## Graphical abstract

A total of 10 Hyperphenylalaninemia patients were enrolled in this study. The coding and adjacent intronic regions of PTS and QDPR genes were examined using Sanger sequencing. Protein modeling was also performed for novel identified mutations.

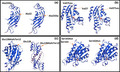

## 1 INTRODUCTION

Hyperphenylalaninemia (HPA) is the most common inborn error of amino acid metabolism across the world. It is caused by a deficiency in the components of the metabolic pathway of phenylalanine (Phe), such as phenylalanine hydroxylase (PAH) or tetrahydrobiopterin (BH4), leading to abnormal accumulation of the Phe in the body fluids and nervous system which followed by problems in the brain and skin (Aliu et al., 2018; Porta et al., 2020; Pronina, 2012). If left untreated, HPA can lead to severe mental retardation. However, by early initiation of the proper treatment, patients can benefit from an average intelligence quotient (IQ) (Flydal et al., 2019; Williams et al., 2008).

Several genetic factors are involved in the development of HPA (Thöny et al., 2000). This condition occurs in 98% of cases due to disease‐causing variants in the *PAH* gene (OMIM accession number: 612349), leading to PAH deficiency that impairs the hydroxylation of Phe and prevents its conversion into tyrosine (Abbaskhanian et al., 2017; Gundorova et al., 2021; Koch et al., 1998). At least 2% of HPA cases are caused by defects in BH4 metabolism. This subtype of HPA, which occurs due to a deficiency in BH4, is called BH4 deficiency, malignant phenylketonuria (malignant PKU), or non‐PKU hyperphenylalaninemia (Ilgaz et al., 2021). These patients may have variable neurologic abnormalities; Some may have a mild or asymptomatic phenotype, and some may have severe brain problems even with the treatment regimen and normalization of plasma Phe levels (Anjema, 2020). Genes such as *QDPR* (OMIM accession number: 612676), *PTS* (OMIM accession number: 612719), *PCBD1* (OMIM accession number: 126090), *GCH1* (OMIM accession number: 600225), *SPR* (OMIM accession number: 182125), and *DNAJC12* (OMIM accession number: 606060) are essential in the biosynthesis or regeneration of BH4, that deficiency in each of these genes leads to BH4 deficiency (Brennenstuhl et al., 2019; Burlina et al., 2022; Himmelreich et al., 2021; Opladen et al., 2020). Unlike the classic forms of BH4 deficiency caused by disease‐causing variants in the genes encoding 6,7‐dihydropteridine reductase (DHPR), 6‐Pyruvoyl‐tetrahydropterin synthase (PTPS), pterin‐4 alpha‐carbinolamine dehydratase 1 (PCD), and GTP cyclohydrolase I (GTPCH) that lead to HPA, BH4 deficiency caused by a defect in SR may not cause the HPA phenotype (Blau et al., 1993; Niu, 2011; Wang et al., 2018). Disorders resulting from disease‐causing variants in all of these genes are inherited autosomal recessively, except for *GCH1*, which is inherited in both autosomal recessive (ARGTPCH deficiency) and autosomal dominant (ADGTPCH deficiency) forms. It is important to note that ARGTPCH deficiency manifests with increased plasma phenylalanine levels, while no change in phenylalanine levels occurs in ADGTPCH deficiency (Himmelreich et al., 2021; Niu, 2011). The most common cause of BH4 biosynthesis deficiency is a defect in the *PTS* gene, which accounts for 65% of BH4 deficiency cases. Defect in the *QDPR* gene also accounts for approximately 25% of cases. The other genes mentioned above cover 10% of BH4 deficiency cases (Cotton & Hyland, 2001; Flydal et al., 2019; Himmelreich et al., 2021). All infants should undergo a newborn screening program (NBS) in the first few days after birth to determine plasma Phe levels and identify cases of HPA (Shi et al., 2012). In Iran, NBS was launched as a pilot in several provinces in 2006 and became a national program in 2011 (Pourfarzam & Zadhoush, 2013; Shokri et al., 2020). The incidence of HPA varies around the world. This rate is estimated at 1 in 10,000 in Europe and 1 in 12,000 live births in the United States. The lowest incidence of HPA is related in Finland and Thailand, with one case per 100,000 and 200,000 live births, respectively. The highest incidence has been reported in Turkey, with one case per 2600, Ireland, with one case per 4500, and Scotland, with one case per 5300 live births (Foreman et al., 2021; Wang et al., 2018, 2021). Koochmeshgi J et al. reported the incidence of HPA as about one case per 3600 live births in Iran, likely due to the high rate of consanguineous marriages. The incidence of BH4 deficiency has remained unknown worldwide and in Iran (Koochmeshgi et al., 2002; Zare‐Karizi et al., 2011).

In the present study, non‐PKU hyperphenylalaninemia patients of Khorasan Razavi province who were referred to Imam Reza and Akbar hospitals in Mashhad were studied, and 10 unrelated patients were selected. In these patients, coding and adjacent intronic regions of *QDPR* and *PTS* genes were examined using the Sanger sequencing method. This study aims to identify disease‐causing variants in HPA patients, which may be helpful in genetic counseling and prenatal diagnosis in patients and their families.

## 2 MATERIALS AND METHODS

### 2.1 Subjects

**FIGURE 1:**
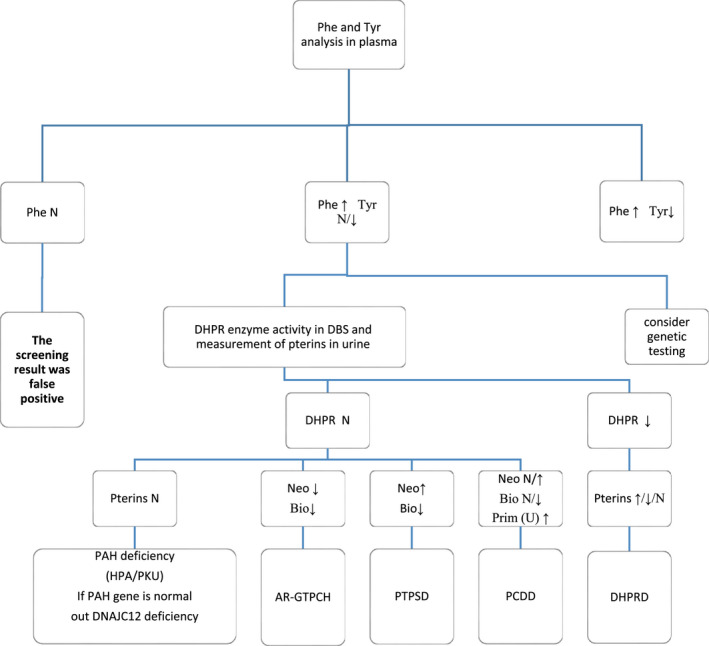
Diagnostic flow chart for hyperphenylalaninemia subtypes in the newborn screening. Bio, biopterin; DBS, dried blood spots; N, normal; neo, neopterin; Phe, phenylalanine; prim, primapterin; Tyr, tyrosine.

From 264 HPA patients referred to Imam Reza and Akbar hospitals in Mashhad, based on the results of biochemical tests, it was determined that 250 of them had PAH deficiency and 14 of them had BH4 deficiency. This study examined 10 of these 14 patients with BH4 deficiency to identify disease‐causing variants in *QDPR* and *PTS* genes. The age of patients ranged from 3 to 18 years. Most of these patients were identified during the NBS program. Patients were classified into BH4‐deficient subtypes based on Phe and tyrosine levels in the blood, biopterin and neopterin levels in urine, and DHPR enzyme activity in dried blood spots (DBS) (Figure 1). Comprehensive explanations were provided to patients and their parents, and all signed an informed consent form for molecular testing. Then, patients' family history was recorded, and pedigrees were drawn for the families by INVITAE online program.

### 2.2 DNA extraction

Five milliliters (ml) of peripheral blood were taken from the patients and their parents in tubes containing EDTA anticoagulants. DNA extraction was performed using a salting‐out method according to the standard protocols in molecular genetics laboratories.

### 2.3 Primer design

Specific primers were designed by the Primer 3 online tool that covered all of the coding and adjacent intronic regions of *PTS* (NM_000317.3) and *QDPR* (NM_000320.3) genes (Table 1). In designing these primers, all essential parameters, such as primer length, product length, GC percentage, annealing temperature, the difference in annealing temperature of primers, etc., were considered. Secondary structure formation was also assessed by the Oligo Analyzer tool in IDT collection. The Primer Blast program from the NCBI database was applied to check primer specificity. Finally, primer synthesis orders were sent to Pishgam Biotechnology Company.

**TABLE 1:** Primers used to sequence coding and adjacent intronic regions of the *QDPR* and *PTS* genes

Exon	Primer	Primer sequence	Tm (°C)	Amplicon (bp)
*QDPR* ^a^ */1*	Forward	GGCCGAAGTTACAGTCCCTC	60	459
	Reverse	GAAACAGGAATAGACGCGTAGAC	59.5	
*QDPR/2*	Forward	ACTACACCTGGCCAACTCC	59	274
	Reverse	ACATACAGCCAAAGGAAGAACATAC	59	
*QDPR/3*	Forward	ATGCTCATCAGAGCTTAGTCTTC	58	345
	Reverse	GCCTGTCACCTAGTGCAAAC	59	
*QDPR/4*	Forward	GTGCTGTTTGTGTTAGACCTTG	58	378
	Reverse	AGGCTTTAACAGTCCTCATCCC	59.5	
*QDPR/5*	Forward	CCCATCCCCTCTTTCTCGTG	60	382
	Reverse	GGAAAGCTACAGTCAGACAAACG	60	
*QDPR/6*	Forward	TTGTCCTGCTGTATGTTTGCG	59.5	355
	Reverse	AGCTACTCTGAGATTCCGTCTG	59	
*QDPR/7*	Forward	TAAACAGTCGCTGCTGTGCC	61	329
	Reverse	CGTACCACAAACAGGGGTTACAG	61	
*PTS* ^b^ */1*	Forward	AGATAGCGAGAGACACCCTTAAC	59	485
	Reverse	CTCCGTTAACCATCAAGCTCC	59	
*PTS/2*	Forward	GATTTCTGACTCTCCCTTTGGTG	59	321
	Reverse	TGTGTCCGTAAGTTTTCCCATTC	59	
*PTS/3*	Forward	TCCTTTGTCTCGATTGTGTCTTG	59	348
	Reverse	GCCAACAATGAAGCAATACTGAC	59	
*PTS/4*	Forward	CAGTCTCTGCACATTGTACTGC	59.5	237
	Reverse	GAGATAACTGGTTGGGGAGGTAG	59.5	
*PTS/5*	Forward	AGTTAGTGGCTAAGTGATAAGGTG	58	398
	Reverse	AAACAGCTACATTTTCAGTCGTG	58	
*PTS/6*	Forward	CTGTATCTTGCCTTATGTGGATTG	58	403
	Reverse	TCACGTGTTGACCTCTTAATATTCC	59	

^a^ NM_000320.3 was used as a reference sequence to design primers for exons 1–7 of the *QDPR* gene.

^b^ NM_000317.3 was used as a reference sequence to design primers for exons 1–6 of the *PTS* gene.

### 2.4 Sanger sequencing

In the first step, the PCR reactions were primed using a 2X Taq PreMix (Pars Tous, Ref. No. 101081, Iran) and suitable primers (10 picomolar) at specific temperature conditions for 35 cycles in a thermocycler machine (SensoQuest GmbH, Germany) only for patients. PCR products were sent to Kawsar Biotechnology Company for Sanger sequencing after electrophoresis on 2% agarose gel and confirmation of PCR product specificity. In the second step, identified variants in patients were confirmed in their parents. All sequencing data were visualized by UGENE software and blasted to reference sequences from the NCBI database. To determine their clinical significance, the identified variants were examined in disease‐related databases such as ClinVar, HGMD, OMIM, and VarSome; if variants were not registered in these databases, they were also checked in population databases such as dbVar, dbSNP, Iranom, and gnomAD to determine their frequency and registration status.

### 2.5 Bioinformatic analysis of novel variants

Novel variants were examined in terms of their frequency in population databases, including dbVar, dbSNP, Iranom, and gnomAD, as well as ClinVar, HGMD, and OMIM databases for clinical significance. Also, the effect of variants on protein structure, function, and stability was predicted through several online tools such as MutationTaster and PROVEAN. It was performed mainly indirectly using the results provided by the VarSome database from more than 10 online tools.

### 2.6 Protein modeling

In this study, the SWISS‐MODEL online program was used to visualize the effect of novel variants on protein structure. The impact of variants on protein structure was assessed using a comparison to the natural form of the enzyme protein. This program requires a protein sequence and a suitable template for protein modeling. The reference protein sequences were obtained from the Uniprot database. To achieve the protein sequences resulting from the effect of novel variants, the cDNA sequences of the reference transcripts were obtained from the NCBI database; after that, the desired change was applied to them, and using the Translate tool of the ExPASy database, they were translated into “amino acid sequence” which from the Open Reading Frames (ORFs) displayed, the correct ORFs were selected by comparing the displayed frames with the reference proteins sequences. To find the most suitable templates for protein modeling, the blastP program of the NCBI database was used, and the amino acid sequence of the proteins was blasted to find the appropriate template in the PDB (Protein Data Bank) database. Of the blasted results, we selected the templates with the most coverage and identity to our interest protein. The selected templates must cover the mutated region of the proteins. The monomeric form of DHPR (PDB ID: 1HDR_A) and PTPS (PDB ID: 3I2B_A) enzymes was used for this purpose.

## 3 RESULTS

### 3.1 Classification of patients

Based on the results of biochemical tests, including measuring the Phe and tyrosine levels in the blood, urinary pterins (neopterin and biopterin), and DHPR enzyme activity on DBS, it was found that four patients have DHPR deficiency and six other patients have PTPS deficiency (Table 2). Also, the pedigree of each family was drawn based on the information received from the families using the INVITAE online tool (Figure S1).

**TABLE 2:** Classification of BH4‐deficient patients based on biochemical data

Patient	Age	Gender	Phe at the time of diagnosis (mg/dl)	Neopterin (mmol/Mol Cr)	Biopterin (mmol/Mol Cr)	DHPR enzyme activity (mU/mg)	When was the disease diagnosed?	Diagnosis
P1	7	Female	7.3	2.18	7.2	0	Diagnosis at the time of screening	DHPRD
P2	8	Female	13.61	1.4	6.23	0	Diagnosis at 20 months (not screened)	DHPRD
P3	3	Male	4	2.78	1.48	0	Diagnosis at the time of screening	DHPRD
P4	18	Female	16.37	11.5	0.57	0	Diagnosis at 1 year of age (not screened)	DHPRD
P5	4	Female	13.7	36.5	0	3.7	Diagnosis at the time of screening	PTSD
P6	4	Male	5.7	14.5	0.03	2.92	Diagnosis at the time of screening	PTSD
P7	3	Male	11.1	8.19	0.9	4.2	Diagnosis at the time of screening	PTSD
P8	10	Male	2.5	7.65	0.03	1.9	Diagnosis at the time of screening	PTSD
P9	9	Female	4.7	14.35	0.32	2.48	Diagnosis at 11 months (not screened)	PTSD
P10	8	Male	5.6	12.31	0.13	4.1	Diagnosis at the time of screening	PTSD

### 3.2 Variant spectrum

Examination of the concentration and purity of extracted DNAs using Nanodrop showed an average concentration of 250–900 ng/μl and a 260/280 ratio between 1.7 and 2, which was suitable for PCR. Electrophoresis on 1% agarose gel also confirmed their proper integrity. The results of Sanger sequencing of all exonic and adjacent intronic regions of the *QDPR* and *PTS* genes led to the determination of disease‐causing variants in the patients. To confirm the inheritance of the variants and perform segregation analysis, the identified variants were also checked in the parents, which showed a heterozygous state in their associated alleles. In this study, 10 patients and a total of 20 alleles were studied, which led to the identification of 10 different variants (Table 3). The clinical interpretation of the genetic variants was investigated using the VarSome database.

**TABLE 3:** The spectrum of variants identified in 10 BH4‐deficient patients

ID	Gene	Exon/intron	Allele 1	Allele 2	Clinical effect
P1	*QDPR* ^a^	Exon 4	c.383_407del(p.Glu128AlafsTer12)	c.383_407del(p.Glu128AlafsTer12)	Pathogenic
P2	*QDPR*	Exon 5	c.488G>A(p.Ser163Asn)	c.488G>A(p.Ser163Asn)	Likely pathogenic
P3	*QDPR*	Exon 1	c.79G>T(p.Val27Leu)	c.79G>T(p.Val27Leu)	Likely pathogenic
P4	*QDPR*	Exon 1	c.68G>A(p.Gly23Asp)	c.68G>A(p.Gly23Asp)	Likely pathogenic
P5	*PTS* ^b^	Intron1‐2 Exon 6	c.84‐3C>G	c.317C>T(p.Thr106Met)	Pathogenic Pathogenic
P6	*PTS*	Exon 1	c.65C>G (p.Ala22Gly)	c.65C>G(p.Ala22Gly)	Likely pathogenic
P7	*PTS*	Exon 2	c.155A>G(p.Asn52Ser)	c.155A>G(p.Asn52Ser)	Likely pathogenic
P8	*PTS*	Exon 5	c.259C>T(p.Pro87Ser)	c.259C>T(p.Pro87Ser)	Pathogenic
P9	*PTS*	Exon 1	c.74G>A(p.Arg25Gln)	c.74C>A(p.Arg25Gln)	Pathogenic
P10	*PTS*	Exon 2	c.155A>G(p.Asn52Ser)	c.155A>G(p.Asn52Ser)	Likely pathogenic

^a^ NM_000320.3 was used as a reference sequence to describe the variants and numbering of the residues.

^b^ NM_000317.3 was used as a reference sequence to describe the variants and numbering of the residues.

The missense variant c.155A>G in the *PTS* gene was strangely identified in two unrelated patients; therefore, it was the most common variant in this gene among the patients. Except for the c.383_407del variant in the *QDPR* gene, a deletion variant, all variants identified in both the *PTS* and *QDPR* genes were missense. Also, in terms of the distribution of variants in the coding and non‐coding regions, except for the c.84‐3C>G variant in the intron 1 of the *PTS* gene, all other variants were located in the exonic regions. Among all the patients studied, except for patient 5, a compound heterozygote, the rest had homozygous variants.

**FIGURE 2:**
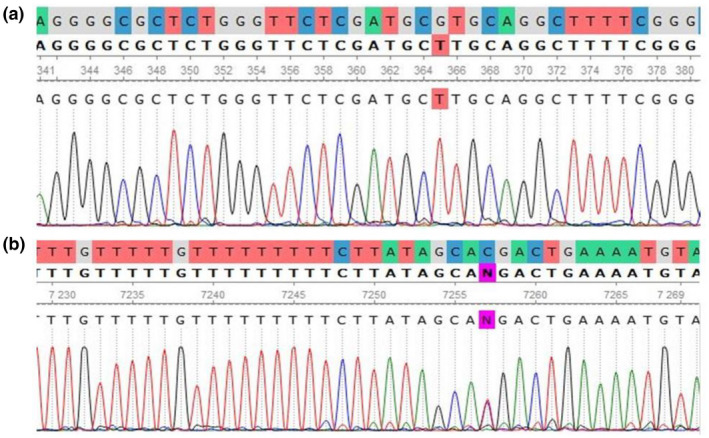
Variant analysis of *QDPR* and *PTS* genes in HPA patients. The reference sequence is at the top of each chromatogram, where each nucleotide is marked with a different color. (a) c.79G>T variant as homozygous in exon 1 of the *QDPR* gene. (b) c.317C>T variant as heterozygous in exon 6 of *PTS* gene.

Of the total variants identified in the *PTS* gene, two variants in exon 2, two variants in exon 1, one variant in exon 5, one variant in exon 6, and one variant in intron 1, and in connection with the *QDPR* gene, two variants in exon 1, one variant in exon 4 and one variant in exon 5 were identified. An example of the identified variants in the *QDPR* and *PTS* genes, visualized by UGENE, is shown in Figure 2.

### 3.3 Novel variants

**FIGURE 3:**
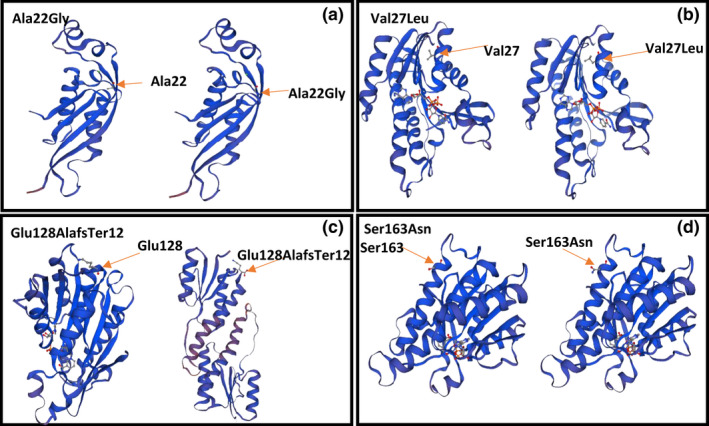
Predicted models for DHPR and PTPS enzymes using the swiss‐prot online tool. (a) Ala22Gly in PTS; (b) Val27Leu in DHPR; (c) Glu128AlafsTer12 in DHPR; (d) Ser163Asn in DHPR.

This study identified three novel variants in the *QDPR* gene, including c.79G>T, c.383_407del, and c.488G>A, and a novel variant c.65C>G in the *PTS* gene. These variants were not recorded in disease‐related databases, and their clinical significance was predicted using online bioinformatic tools such as SIFT, MutationTaster, PolyPhen‐2, etc., provided collectively in the VarSome database. We submitted these novel variants in the ClinVar database, which are now accessible with accession ID SCV002503585, SCV002505653, SCV002505654, and SCV002505655. We also performed protein modeling for novel variants using the Swiss‐Model online tool. The monomeric form of DHPR (PDB ID: 1HDR_A) and PTPS (PDB ID: 3I2B_A) enzymes was used as templates in this process. Variant c.79G>T leads to the replacement of valine with leucine at position 27, variant c.488G>A shows the replacement of serine with asparagine at position 163, and c.383_407del leads to a frameshift variant, causing the replacement of glutamate with alanine at position 128 and premature termination of translation at 12 amino acids after variant site that is resulting in a truncated DHPR protein; also the variant c.65C>G leads to replacement of alanine with glycine at position 22 in PTPS protein. The predicted models are shown in Figure 3.

## 4 DISCUSSION

According to the ACMG classification, genetic variants are divided into four groups: pathogenic, likely pathogenic, uncertain significance, likely benign, and benign. The evidence of pathogenicity used to introduce the variants as pathogenic or likely pathogenic is placed in four groups: very strong (PVS1), strong (PS1‐4), moderate (PM1‐6), and supporting (PP1‐4). Finally, a combination of evidence is used to determine the variant's pathogenicity (for more details on the rules for combining the criteria for grouping sequence variants, please see Table 5 of the ACMG guidelines at https://www.acmg.net/docs/standards_guidelines_for_the_interpretation_of_sequence_variants.pdf).

In this study, two different variants in exon 1 of the *QDPR* gene were identified in two patients (P3 and P4). In one of these patients (P3), the c.79G>T variant occurred homozygously, leading to the replacement of valine with leucine at position 27 of the DHPR protein. This missense variant appeared in the NADH domain of the DHPR protein. This variant is considered likely pathogenic in the ACMG classification (PM1, PM2, PM5, PP1, and PP3). The population frequency of this allele is very low; its frequency has been reported in the gnomAD population project in two populations of Europe and South Asia, which are 0.00000957 and 0.0000658, respectively. Its average population frequency in this population project is estimated at 0.0000126. The very low frequency of this variant further confirms its pathogenicity.

Leucine is a more non‐polar amino acid than valine; therefore, replacing valine with leucine can affect the folding and interactions of DHPR protein with other factors. Variant effect prediction with 12 predictor software including BayesDel_addAF, DANN, DEOGEN2, EIGEN, FATHMM‐MKL, LIST‐S2, M‐CAP, MVP, MutationAssessor, MutationTaster, PrimateAI, and SIFT, all predicted damaging this variant that this would provide further confirmation for its pathogenicity. It is worth noting that this variant was not registered in the ClinVar and other clinical databases; no information about it was available in the literature reviews. This novel variant was registered in the ClinVar with the submission name SUB11371973.

In another patient (P4), the c.68G>A variant was identified as homozygous in the *QDPR* gene. This missense variant converts glycine (a non‐polar amino acid) to aspartate (a polar amino acid) at position 23 of the DHPR protein. The replacement of a negatively charged amino acid in the position of non‐polar amino acid changes the correct folding of the protein and likely affects its function. Like the c.79G>T variant, this variant occurs at the NADH binding site in the DHPR protein. This variant is considered likely pathogenic in the ACMG classification (PM1, PM2, PP1, PP3, and PP5). The population frequency of this variant in the gnomAD population project is estimated at 0.0000242 and 0.0000147 in African and European populations, respectively. The average population frequency is estimated to be 0.0000132, which is a very low population frequency and is a confirmation of the pathogenicity of this variant.

In 1993, Smoker et al. confirmed the occurrence of this variant in *E. coli* and showed that this variant significantly reduces enzyme function. They claimed that this variant was common in the populations of Mediterranean regions (Smooker et al., 1999). In 1998, Irma Dianzani et al. showed that the c.68G>A variant affects the NADH binding site in the N‐terminal DHPR protein and is associated with a severe phenotype in homozygotes (Dianzani et al., 1998).

In exon 4 of the *QDPR* gene, a 25‐nucleotide deletion variant of c.383_407del was identified as homozygous in one of the patients (P1). This deletion is a frameshift variant that replaces glutamate with alanine at position 128 of the DHPR protein. The protein sequence after the variant site is not similar to the main protein and causes the protein to shorten and terminate prematurely at 12 amino acids after position 128. The variant is considered pathogenic in the ACMG classification (PVS1, PM2, and PP1). This loss of function variant occurs in the highly conserved region of the protein and results in the formation of a null allele, resulting in a lack of expression of the corresponding protein. This variant was not reported in any population in the gnomAD population project. Variant effect prediction using phyloP software further confirmed its pathogenicity. This novel variant was registered in the ClinVar with the submission name SUB11406382.

The c.488G>A variant in exon 5 of the *QDPR* gene was identified in another patient (P2). This missense variant converts serine to asparagine at position 163 of the DHPR protein. This variant occurs in a highly conserved region of the protein. Although this substitution leads to the replacement of one polar amino acid with another polar amino acid, asparagine is larger and less hydrophobic than serine; therefore, it can intervene in the interaction of the protein with other molecules or other parts of the protein, resulting in the formation of a damaged protein. Serine also forms a hydrogen bond with glutamine at position 159, which asparagine cannot create due to differences in size. This variant is the likely pathogenic in the ACMG classification (PM2, PP1, PP2, and PP3). On the other hand, in the gnomAD population project, it has not been reported in any population. Variant effect prediction using 13 predictor software confirmed its pathogenicity. This evidence indicates that this variant is pathogenic. This novel variant was registered in the ClinVar with the submission name SUB11406386.

Regarding the *PTS* gene, two different variants were identified in exon 1 of two patients (P6 and P9). The variant c.65C>G, identified as homozygous in one of the patients (P6), resulted in the conversion of the alanine to glycine at position 22 of the PTPS protein. Although one non‐polar amino acid is replaced by another non‐polar amino acid, glycine is smaller than alanine (the smallest amino acid); thus, a change in the size of an amino acid in a particular position can affect the interactions that naturally occur between that position and other molecules or other parts of the same protein. It can also interfere with the proper folding of the protein, which may cause it to malfunction and change its stability. This variant has not been reported in any population in the gnomAD population project. Variant effect prediction using 11 predictor software including BayesDel_addAF, DANN, DEOGEN2, EIGEN, FATHMM‐MKL, LIST‐S2, M‐CAP, MVP, MutationAssessor, MutationTaster, PrimateAI, and SIFT, all confirmed it as likely pathogenic variant according to the ACMG classification (PM1, PM2, PP1, PP2, and PP3). There is no information about this variant in the ClinVar and other clinical databases or literature reviews. This novel variant was registered in the ClinVar with the submission name SUB11406390.

The c.74G>A variant was another variant identified in exon 1 of the *PTS* gene (P9). This homozygous variant converts arginine to glutamine at position 25 of the PTPS protein. Although both amino acids are non‐polar, they are of different sizes and, therefore, can impair protein activity or stability. This change occurs at the zinc ion binding site, which can be affected by this replacement. UniProt and ClinVar databases classified it as a pathogenic variant based on functional studies. This variant places in a similar classification based on the ACMG guideline (PS3, PM1, PM2, PM5, PP1, PP2, and PP3). In addition, the conversion of arginine to another amino acid (glycine) has already been reported as pathogenic in the UniProt and ClinVar databases, indicating the importance of arginine at this site. This variant has not been reported in any population in the gnomAD population project, which further reinforces its pathogenicity. Variant effect prediction using predictor software, except in one case, all predicted the pathogenicity of this variant. By analyzing *PTS* gene variants in 1998 in Chinese patients with hyperphenylalaninemia due to BH4 deficiency, TT Liu et al. showed that approximately 5.3% of patients with a defect in *PTS* showed this variant, which was reported as a novel missense variant mainly in the patients with severe clinical symptoms (Liu et al., 1998). In 1994, B Thöny et al. showed that expression of this mutant allele in combination with the natural allele results in a 14% residual enzyme activity. They reported that the variant occurs in an evolutionarily conserved nucleotide that reduces enzyme activity, resulting in BH4 deficiency (Thöny et al., 1994).

The c.155A>G variant was identified as homozygous in exon 2 of the *PTS* gene of two patients (P7 and P10). This variant replaces asparagine with serine at position 52 of the PTPS protein. Asparagine is a partially positively charged polar amino acid replaced by serine, a negatively charged polar amino acid that can impair protein function. Asparagine 52 is located in the highly conserved region of the protein, close to the zinc ion binding site, where any change in it can affect this region and impair the protein's function. UniProt and ClinVar, both jointly and based on previous studies, considered this variant pathogenic, but in the ACMG classification, this variant is grouped as likely pathogenic (PM2, PP1, PP2, PP3, and PP5). Variant effect prediction using 10 predictor software that assigned this variant pathogenic, in contrast to the two other software that predicted it as a benign variant, further confirmed its pathogenicity. This variant has been reported in the gnomAD population project in East Asian and European populations with a frequency of 0.00147 and 0.0000176, respectively, and an average population frequency of 0.000116 confirm the pathogenicity of this variant. Chiu YH and colleagues 2012 conducted a study on the spectrum of *PTS* founder variants in the East Asian population, identifying the c.155A>G variant as one of the most common disease‐causing variants in the *PTS* gene (Chiu et al., 2012). TT Liu in 2001 and Wang X and colleagues in 2019 identified this variant as one of the most abundant disease‐causing variants in the *PTS* gene in Xiamen, China (Liu, Chiang, et al., 2001; Wang et al., 2019).

The c.84‐3C>G variant was identified between exons 1 and 2 (introns 1–2) of the *PTS* gene in one patient (P5) as a compound heterozygote (another allele was c.317C>T). This variant is located near the receptor splice site in intron 1; therefore, it is considered a splicing variant that leads to a loss of function in the corresponding protein. Based on performed studies, this variant is considered pathogenic in the ClinVar database (Buratti et al., 2007; Leuzzi et al., 2010; Oppliger et al., 1997; Romstad et al., 1999; Zhang, 1998). This variant has a similar status in the ACMG classification (PP3, PP5, Pm2, and PP1). This variant affects the highly conserved region of the protein at the zinc ion binding site, which justifies its loss of function. This variant has not been reported in the gnomAD population project, reinforcing its pathogenicity.

The c.259C>T variant was identified as homozygous in exon 5 of the *PTS* gene in one patient (P8). This variant replaces proline with serine at position 87 of the PTPS protein. Replacement of proline, a non‐polar amino acid, with serine, a polar amino acid, alters the protein charge and impairs protein function. The location of this variant is in a highly conserved region of the protein, close to the active site, which increases the possibility of its pathogenicity. UniProt and ClinVar have considered this variant pathogenic based on performed studies (Blau et al., 2000; Chiu et al., 2012; Chloe et al., 2011; Dudesek et al., 2001; Gu et al., 2009, 2014; Hanihara et al., 1997; Imamura et al., 1999; Liu et al., 1998; Liu, Chang, et al., 2001; Liu, Chiang, et al., 2001; Liu & Hsiao, 1996; Oppliger et al., 1997; Romstad et al., 1999; Scherer‐Oppliger, Leimbacher, et al., 1999; Scherer‐Oppliger, Matasovic, et al., 1999; Thöny et al., 1994; Vatanavicharn et al., 2009; Wang et al., 2019; Xiong et al., 2015). This variant is also pathogenic in the ACMG classification (PP5, PM2, PM5, PP1, PP2, and PP3). The conversion of proline to leucine at the same position has already been described in the UniProt database as a pathogenic variant. It has been confirmed by the ACMG, which further confirms the pathogenicity of this variant. Variant effect prediction using predictor software showed that the variant was pathogenic by eight software versus benign by one software. Examination of the frequency of this variant in the gnomAD population project also showed that this variant was reported only in the East Asian population, and its frequency was 0.000384. Its average population frequency is 0.0000131, which is very low and consistent with its pathogenic nature.

The c.317C>T variant has been identified as a compound heterozygote in exon 6 of the *PTS* gene in one of the patients (P5). This variant replaces threonine with methionine at position 106 of the PTPS protein. Threonine is a polar amino acid, the replacement of which with methionine, a non‐polar amino acid, can have a detrimental effect on protein activity. UniProt and ClinVar databases reported this variant as pathogenic based on performed studies (Ashida et al., 1994; Blau et al., 2000; Dudesek et al., 2001; Hanihara et al., 1997; Leuzzi et al., 2010; Liu et al., 1998; Liu, Chang, et al., 2001; Liu, Chiang, et al., 2001; Liu & Hsiao, 1996; Oppliger et al., 1995, 1997; Romstad et al., 1999; Scherer‐Oppliger, Leimbacher, et al., 1999; Scherer‐Oppliger, Matasovic, et al., 1999; Thöny et al., 1994; Ye et al., 2013). This classification is consistent with the ACMG classification (PP5, PM2, PP1, PP2, and PP3). This variant occurs at a highly conserved region in the protein structure. The frequency of this variant in the gnomAD population project and African, Finnish‐European, and non‐Finnish European populations has shown 0.0000487, 0.000679, and 0.0000736, respectively. The average population frequency is 0.0000925, a very low frequency that reinforces other findings that this variant is pathogenic.

In addition to the advantages of our study, it also had some limitations. One of the main limitations is the lack of access to samples from other centers and provinces. With the availability of more samples, it would be possible to identify more common disease‐causing variants in these patients at the country level, and probably more novel disease‐causing variants would be identified; it is also possible to check the distribution of variants in different population subgroups. Because this study was a student thesis, one of its other limitations was the limitation of time and resources, which made further work impossible.

## 5 CONCLUSION

This study investigated disease‐causing variants and how they are distributed in the *QDPR* and *PTS* genes in Khorasan Razavi population. These two genes are involved in the biosynthesis and regeneration of the BH4, which is necessary for the proper function of the PAH enzyme. Disease‐causing variants in them cause HPA. Four different variants in the *QDPR* gene and six different variants in the *PTS* gene were identified in the study. Identification of unregistered variants c.79G>T (p.Val27Leu) and c.383_407del (p.Glu128AlafsTer12) in the BIOPKU database indicates different disease‐causing variants in the *QDPR* gene in the Iranian population compared to European and other Asian people.

Despite the general genetic similarities of our populations in the spectrum of variants, differences were observed with other Asian and European people; As a result, similar but more extensive studies are needed to investigate the distribution of disease‐causing variants in genes involved in non‐PKU hyperphenylalaninemia such as *QDPR*, *PTS*, *PCBD1*, *GCH1*, *SPR*, and *DNAJC12* in the Iranian population. Increasing our awareness of how variants are distributed in the population can pave the way for faster diagnosis and the development of new therapies for this disease.

## AUTHOR CONTRIBUTIONS

Tayebeh Hamzehlouei and Peyman Eshraghi were involved in conception and revision for important intellectual content. Seyedeh Helia Sadat Fatemi, Mahmoud Ghanei, Tayebeh Hamzehlouei, and Peyman Eshraghi were involved in interpretation or analysis of data. Seyedeh Helia Sadat Fatemi and Mahmoud Ghanei were involved in preparation of the manuscript. Tayebeh Hamzehlouei was involved in supervision.

## FUNDING INFORMATION

The Vice Chancellor for Research funded this study at Mashhad University of Medical Sciences by no. 15062.

## CONFLICT OF INTEREST

The authors declare that they have no competing interests.

## ETHICS STATEMENT

This study was approved by the Ethical Committee of Mashhad University of Medical Sciences with reference number IR.MUMS.MEDICAL.REC.1398.332.

## Supporting information


Figure S1

## Data Availability

The data that support the findings of this study are available from the corresponding author upon reasonable request.
