# Supplementary material for: Genetic evaluation of hyperphenylalaninemia patients with tetrahydrobiopterin deficiency in Iranian population: Identification of four novel disease‐causing variants
Source: Mol Genet Genomic Med. 2022 Nov 16;10(12):e2081. doi: 10.1002/mgg3.2081 (PMC9747554; doi:10.1002/mgg3.2081)
Supplement: Supplementary file 1 — Figure S1 [file MGG3-10-e2081-s001.docx]

**
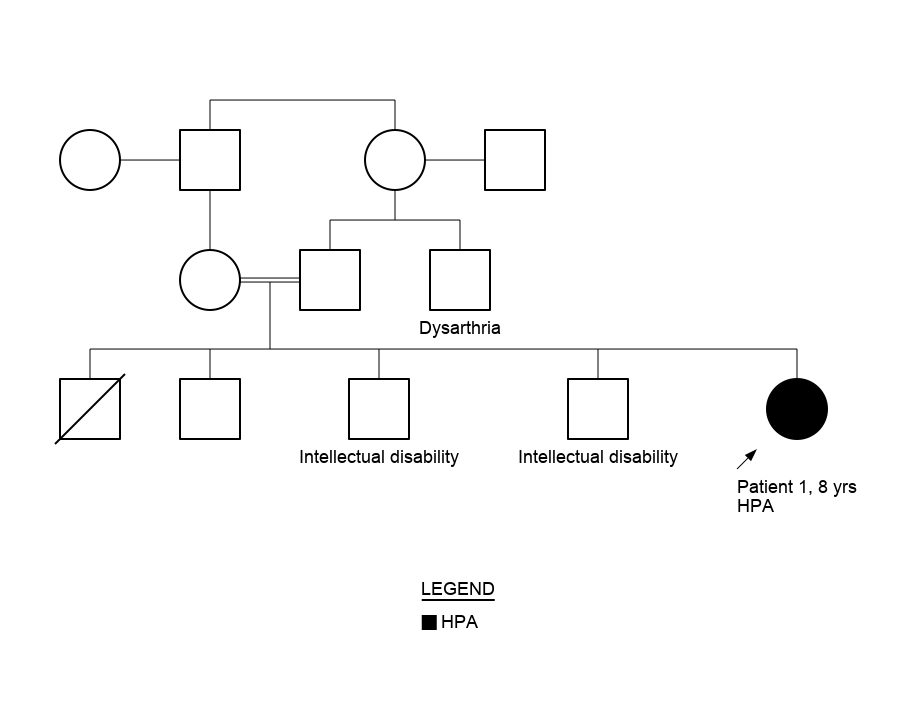
Supplementary Figure 1:** Pedigrees of 10 families with at least one HPA patient

Pedigree Family No. 1


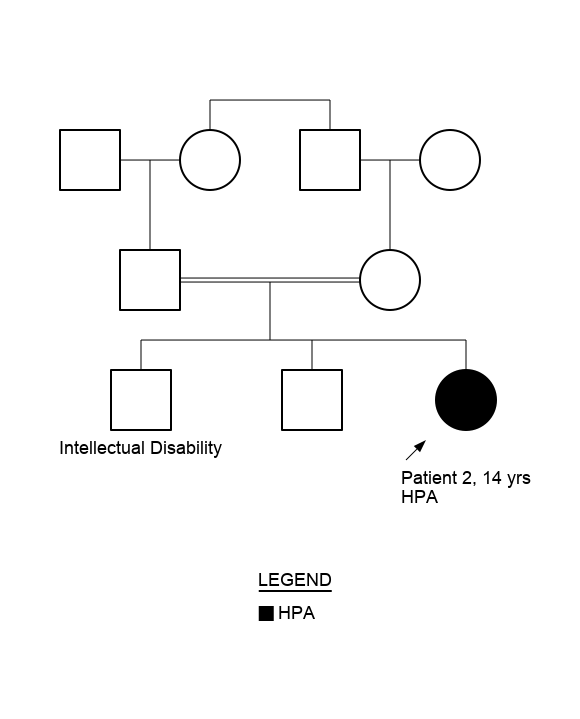


Pedigree Family No. 2


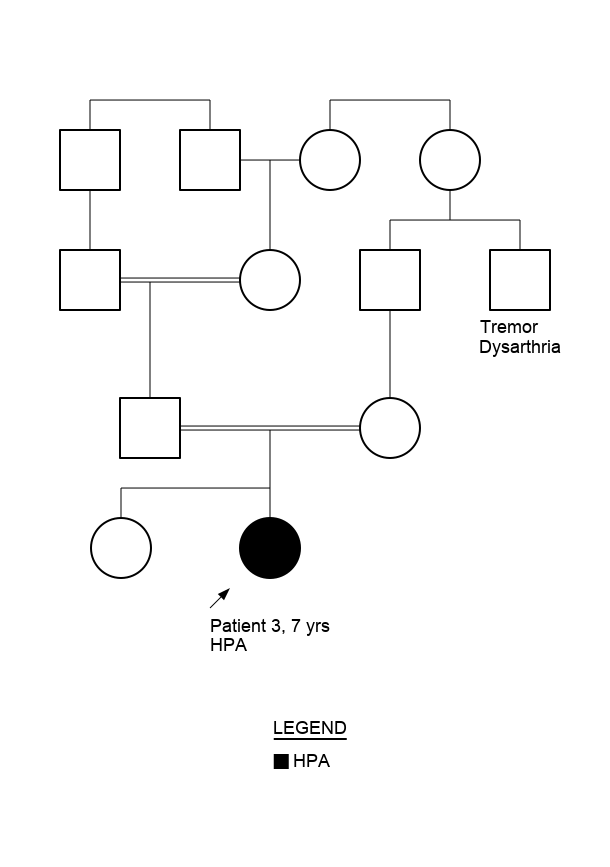


Pedigree Family No. 3


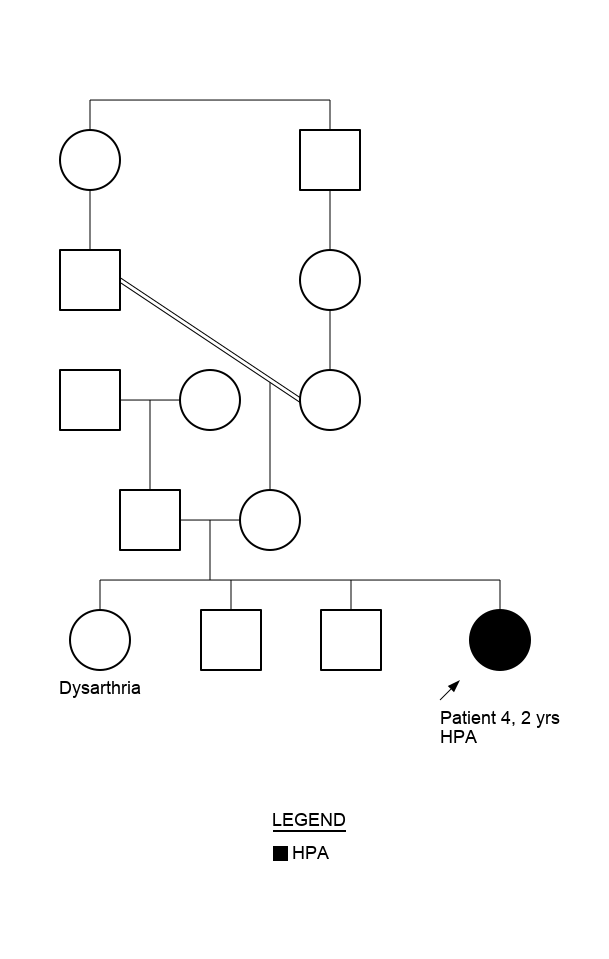


Pedigree Family No. 4


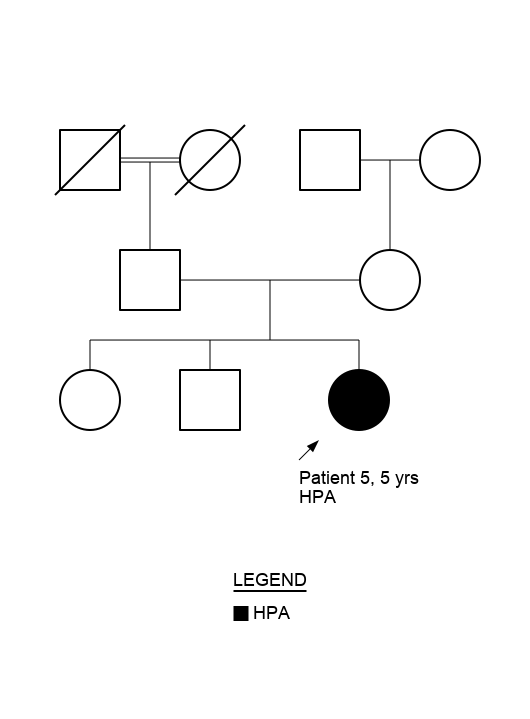


Pedigree Family No. 5


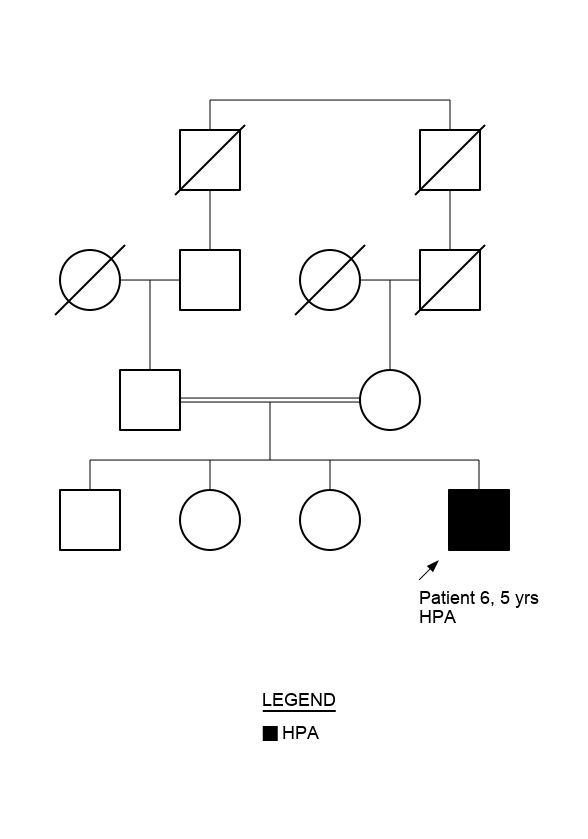


Pedigree Family No. 6


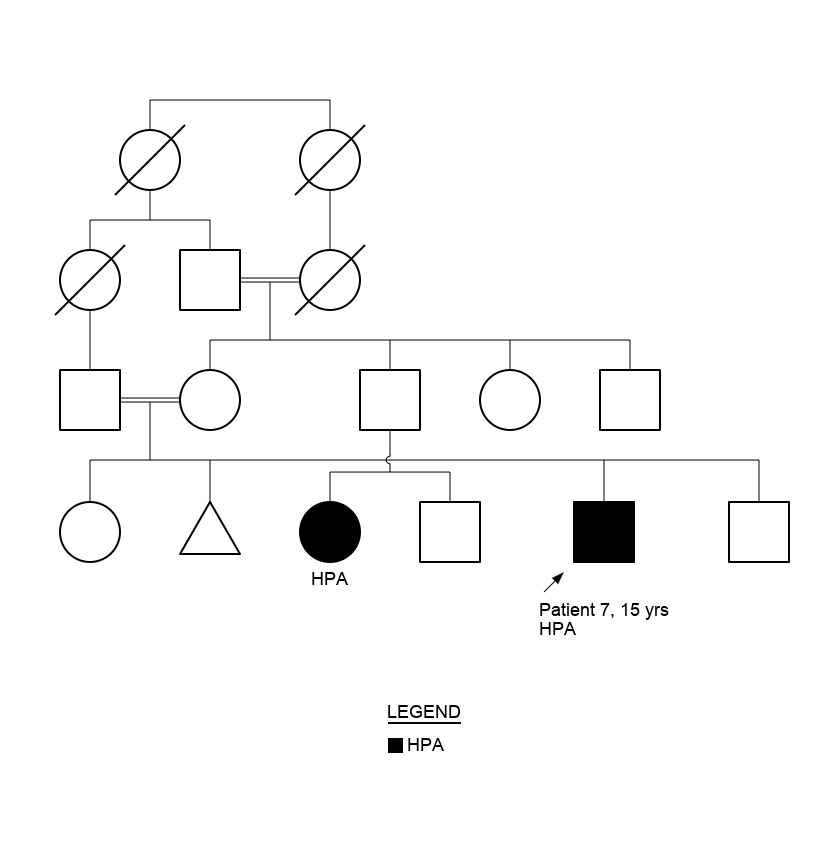


Pedigree Family No. 7


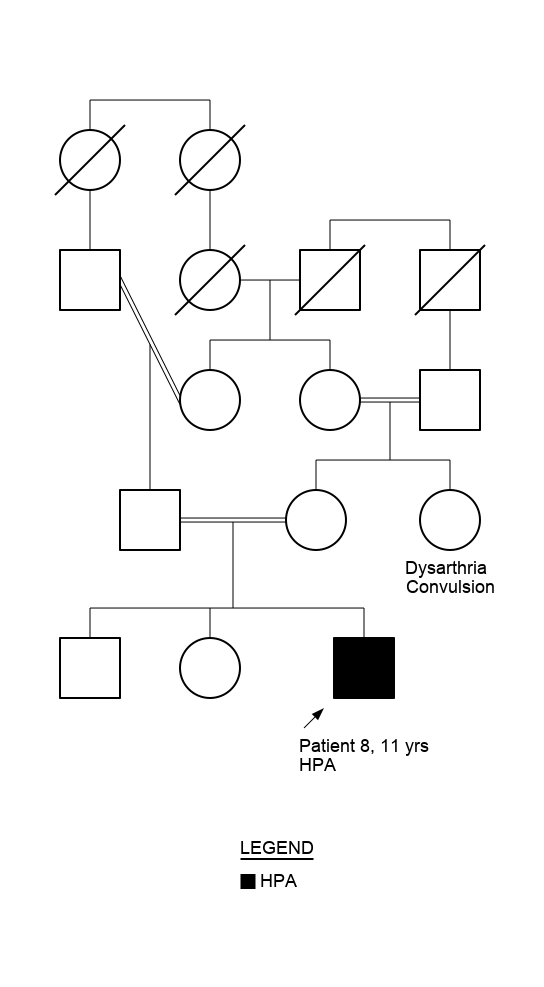


Pedigree Family No. 8


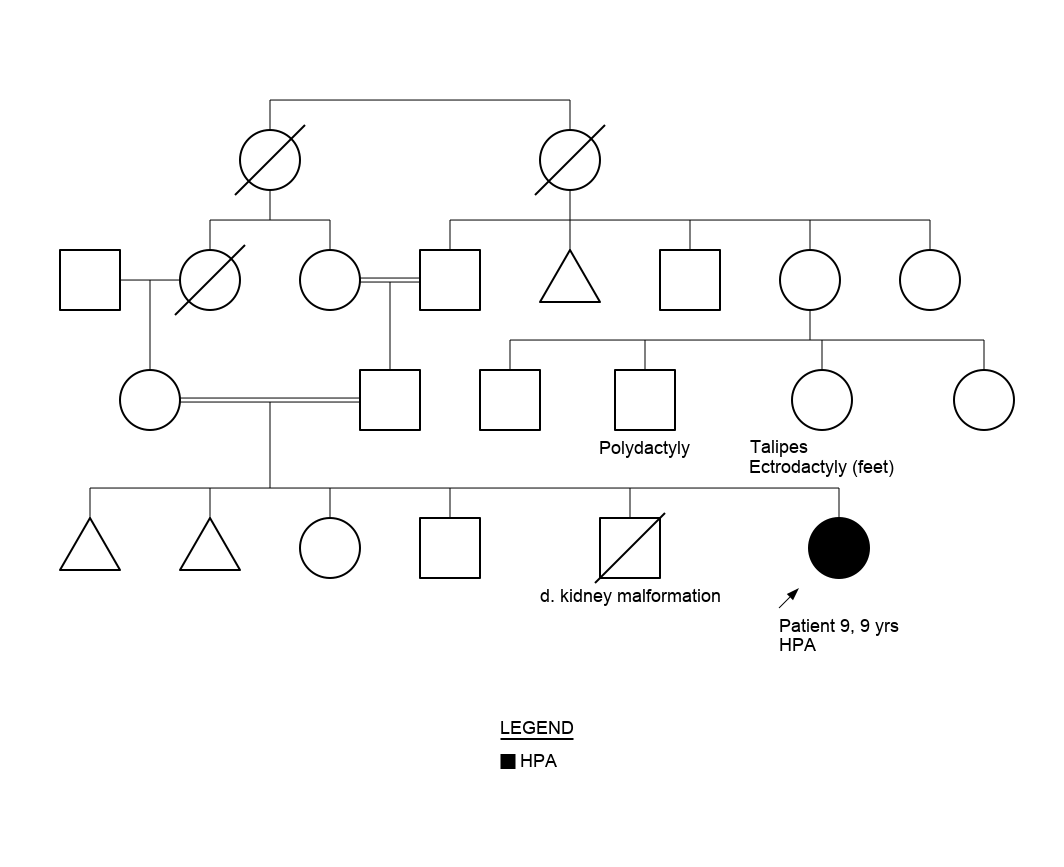


Pedigree Family No. 9


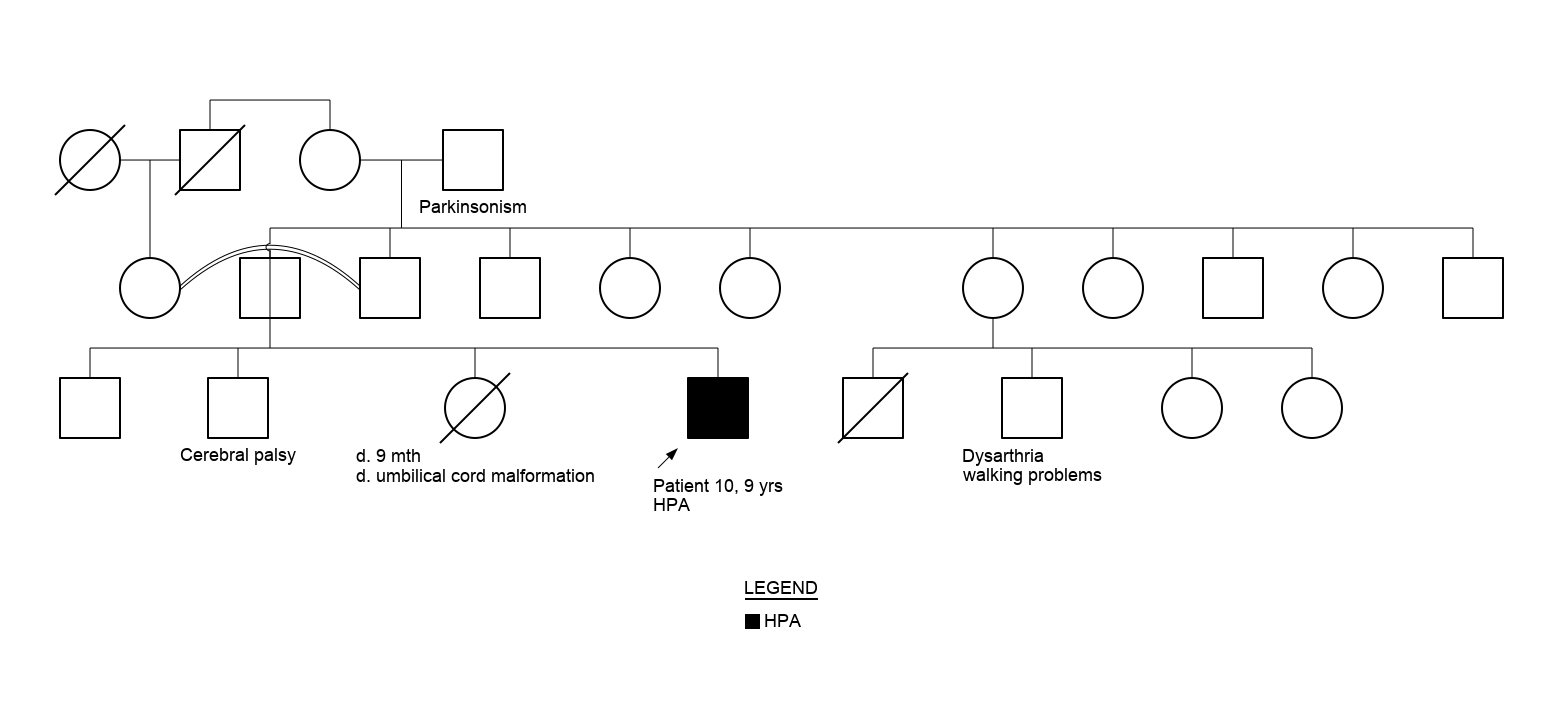


Pedigree Family No. 10
